# Modern Sociology

**Published:** 1900-01-20

**Authors:** 


### MODERN SOCIOLOGY.

### SOME STRANGE FORMS OF DRUNKENNESS.

By a Correspondent.

Our temperance reformers as a rule devote their attention to the denunciation of the commoner forms of alcohol—beer, whisky, champagne. We have heard one earnest apostle speak in fervent terms of the dangers that attend the giving of claret-cup at a dance. But while the evils wrought by these liquors meet us every day, we are apt to ignore the fact that inebriety may result from the consumption of things of which we take no heed.

Ether inebriety is a case in point. The habit of ether-drinking is the direct result of a temperance crusade. In 1838 an earnest and sincere Catholic priest, Father Mathew, began to preach total abstinence in Ireland. Within three years five millions of his fellow-countrymen had joined his banner, and pledged themselves not to touch alcohol. In Great Britain and America the success of his campaign was equally great. But it so happened that between 1842 and 1845 some of the father's converts, residing in a village called Draperstown, in County Londonderry, missing their accustomed stimulant, asked a local medical man to give them something, the consumption of which would not violate their vow of abstention from alcohol, yet which would relieve them of their craving for it. He gave the applicants a drachm of ether in water. The habit spread, till in some of the neighbouring places there was a shop for the sale of ether for every 23 of the population. From that time until now the habit of ether drinking has gone on in that part of Ireland, and men and women, young and old, are alike its victims. Cases have been known elsewhere, but chiefly among those who feel a craving for some stimulant, and do not like to indulge in the ordinary forms of alcohol.

Akin to this habit is that of drinking eau-de-cologne. This practice is, as might be surmised, commoner among women than among men. It was long before it was realised that the taking of a few drops of the perfume on a lump of sugar was in any way a dangerous habit. In older books on the toilet one may find it recommended as tending to make the eyes bright. But there can be no doubt that the habit grows until it becomes absolutely a form of the drink craving. The temptation to indulge in this way is easily comprehensible. The perfume can be easily purchased, and no suspicion is aroused. It may be kept on one's dressing-table without bringing on one's character the stigma that would soon be affixed if a bottle of brandy took its place. A lavish use of the scent externally will conceal the fact that one's very breath is heavy with the odour. It forms, in fact, a conspicuous temptation to those who feel a craving for some stimulant, yet will not, for pride or shame, indulge in the ordinary forms of alcohol. Occasionally a person who has been a victim to inebriety appears to make a rapid cure, and will assure his friends that he—or more frequently she—never touches any form of stimulant. Yet she may be indulging continually in the drinking of eau-de-cologne; and is hardly conscious herself that the new stimulant is as dangerous as the old one. This is a kind of inebriety that offers peculiar temptation to neurotic women.

Liquids that are even less open to suspicion than the scented spirit have yet their victims, who certainly suffer from a kind of inebriety, and are assuredly the victims of a habit difficult to overcome. What would a temperance association say if one of its members were accused of tea or coffee inebriety? Yet the too great use of either of these produces symptoms which are akin to those induced by alcoholic indulgence. The exhilarating effects of tea and coffee are known, and are never objected to, but over-indulgence is followed, not indeed by the violence and stupor of alcoholic intoxication, but by physical evils almost as marked. The nervous system is affected, there is insomnia, much indigestion, and often the sight and hearing are affected. In one case recorded in the annals of the American Society for the Study of Inebriety, absolute delusions of a mild type were noticed in three old women who indulged in excessive tea-drinking. When they were kept from their accustomed indulgence the delusions stopped. In most cases of tea and coffee inebriety, however, the patient not only drank the infusions but ate coffee berries and tea-leaves. The effect of this indulgence is absolutely poisonous. Moreover, the mechanical irritation caused by the presence of the tea or coffee in the bowel not only causes constipation, but affects the nervous system as well. It has even been known to cause delirium, differing very little in its symptoms from delirium tremens.

It is hardly necessary to speak of indulgence in drugs, a habit to which alcoholic drunkards often resort by way of curing their besetting sin, but which may be contracted through the use of the drug, whether it be narcotic or anæsthetic, under medical advice. Most doctors, at least in this country, are so conscious of the risk of establishing a habit of indulging in the steady consumption of a drug first administered in emergency, that they are very chary of affording the relief it may give, even when the patient is suffering severely. In America, where the detestation of alcoholic intoxication is much more intense than it is in this country, there seems to be much less hesitation about the employment of drugs, nor is there the same shrinking from narcomania as a peculiarly insidious and offensive form of indulgence which English people feel. Indulgence in opium, in cocaine, or in chloroform have all their victims, and it has been noticed that indulgence in such drugs is rather common in those prohibition States where alcohol cannot legitimately be procured. Women also, and men whose position is such as to make it peculiarly disgraceful for them to be seen under the influence of liquor, or even to be known to take alcohol, are the most likely to indulge in this way. It is strange and sad that medical men, who know the risks they run, occasionally fall victims to this habit.

The moral, if one may venture to draw one, seems to be this—that the desire for some form of stimulant or another seems to be almost universal, and that merely to withhold opportunity for indulging in one or two special kinds will not cure the craving, though it may prevent some from acquiring it. The cure of inebriety demands active moral and physical effort, and at the best we must be prepared for the morbid desire breaking out in some new form. And one may also infer that their condition is the safest who can exercise the most complete control over their appetite, whether the control takes the form of total abstention or simple moderation.

